# Traditional Practices Used for the Infertility Treatment Among Females in Albaha City

**DOI:** 10.1155/nrp/6934650

**Published:** 2025-09-03

**Authors:** Amnah S. Alghamdi, Hawa Alabdulaziz, Wafaa A. Rashad

**Affiliations:** ^1^Nursing Administration, King Faisal Medical Complex, Taif, Saudi Arabia; ^2^Faculty of Nursing, King Abdulaziz University, Jeddah, Saudi Arabia; ^3^Obstetric and Gynecology Nursing, Faculty of Nursing, King Abdulaziz University, Jeddah, Saudi Arabia

**Keywords:** herbs, infertility, reproductive system, Saudi Arabia, traditional practices

## Abstract

**Background:** Infertility is a reproductive disorder affecting either the male or female reproductive system, characterized by the inability to achieve pregnancy after 12 months or more of regular, unprotected sexual intercourse. It may result from male, female, or idiopathic factors, with certain etiologies being preventable. Management of infertility frequently involves assisted reproductive technologies, including in vitro fertilization (IVF).

**Objective:** This quantitative cross-sectional study aimed to discover the traditional practices used for infertility treatment among females in Albaha City.

**Materials and Methods:** This study was conducted with 251 infertile women between the ages of 50 or beneath who had primary or secondary infertility because of personal reasons or male infertility in the maternity outpatient departments of Albaha City hospitals in Saudi Arabia. The study period lasted from May 30, 2021, until January 2022. The research obtained approval from the Faculty of Nursing ethical committee at King Abdulaziz University Jeddah before participants granted their consent.

**Results:** A total of 251 participants indicated that 51.0% found traditional infertility treatment successful and 45.8% had no previous knowledge about the approach, while 3.2% viewed it negatively. Among the participants, 39.5% revealed that they got pregnant after using traditional treatments, whereas 60.5% said they did not get pregnant. About 29.0% of women who got pregnant achieved it within 6 months and 11.0% of them conceived over 6 months to 1 year. Two-thirds of participants reported no side effects from traditional healing treatment, and half of the respondents planned to repeat their experience. The research conducted on pregnancy and herb consumption demonstrated a lack of statistical importance expressed through the data values (*p* > 0.05).

**Conclusion:** Many participants believed in using traditional practices and visiting traditional healers instead of attending medical facilities and the care services provided by health professionals due to their actions on physiological systems and low cost. Further investigation is required to support the outcomes of this study.

## 1. Introduction

A disorder of the male or female reproductive system known as infertility is characterized by the inability to conceive after 12 months or more of frequent, unprotected sexual activity [1]. Millions of people are affected by infertility, which also affects their families and communities. Global estimated suggested that around 186 million individuals and nearly 48 million couples live with infertility.

According to estimates, one in every six persons of reproductive age will experience infertility at some stage. There are two types of infertility. Infertility qualifies as primary when someone who has never given birth fails to conceive after a year of consistent efforts except when the person is older than 35 years old (and attempts six months instead of 12 months to conceive). Secondary infertility represents when conception fails to occur after achieving a previous pregnancy [2]. Infertility has been an unresolved issue in human reproduction [3, 4].

Infertility in women may result from uterine disorders, ovarian disorders like polycystic ovarian syndrome (PCOS) and other follicular disorders, and endocrine system disorders that lead to hormonal imbalances, including tubal disorders like blocked fallopian tubes and untreated sexually transmitted infections (STIs) or sexually transmitted diseases (STDs), and complications of unsafe abortion, postpartum sepsis, and abdominal or pelvic surgery [5]. In contrast, infertility in men may result from any of these causes, blockage of the reproductive system leading to abnormalities in semen ejection. Semen-carrying tubes (such as ejaculatory ducts and seminal vesicles) may become blocked due to this condition [5].

Infertility affects marital relationships and can lead to psychological issues, making it difficult to access treatment. The prevention, diagnosis, and treatment of infertility are all included in fertility care [6]. In most nations, particularly low- and middle-income nations, obtaining equal and fair access to reproductive healthcare remains difficult. Rarely do national universal health coverage benefit packages prioritize fertility care [7]. Although there are many infertility treatments, most cannot be used due to the high cost of care and the unavailability of resources [8]. In some instances, notwithstanding the use of all possibilities, the success rate of the treatment is not significant.

The first step in treating infertility is a clinical evaluation and examination. The treatment path will rely on previous treatments' results, including the number of pregnancies and births [9]. However, there is still a substantial possibility of early birth, problems throughout pregnancy, and abortion [10]. As a result, some infertile couples choose traditional approaches to therapy [5]. The most prevalent area of additional and alternative medicine is herbal medicine. People utilize it because of its accessibility, low cost, acceptance of the patient's religion and culture, and safety as long as no research has shown any risks [11].

The majority of traditional practices vary from culture to culture throughout the world [12]. In the Arabian country, traditional therapies are frequently employed to treat various ailments, including premenstrual syndrome (PMS) and the symptoms of menopause [13]. Herbs and herbal medicines are important for maintaining health and well-being and preventing illness. Herbs are utilized in conventional medicine to treat a variety of diseases. Additionally, the demand for herbal treatment has grown dramatically globally in the past few years because herbs can enhance fertility and induce pregnancy. However, limited information is available regarding the herbal treatments Saudi Arabian women use to conceive [14].

Therefore, the objective of this quantitative cross-sectional study is to discover the traditional practices used for infertility treatment among females in Albaha City.

## 2. Literature Review

Infertility is a neglected problem in most developing countries because it is not a disease that leads to death. However, it negatively affects the infertile person [15]. Nursing and midwifery education should cover preconception care guidelines for infertile men, women, and couples [16]. Preconception care is the term used to describe interventions given to women, men, and couples before conception. These interventions may be emotional, behavioral, biological, or physical. It attempts to boost women's health and pregnancy outcomes [16].

In order to properly educate and raise awareness, nurses and other health professionals in their sector should be familiar with the traditional techniques employed by couples and the positive and negative effects of such practices [17]. Nurses need to be aware of the emotional needs of couples during the time of treatment, must constantly monitor their temperature, and instruct them about their sex life [18]. Couples can learn about traditional methods of treating infertility, the factors influencing these approaches, and their advantages and disadvantages and can be directed to the right resources by learning about practices [17]. Pharmacological surveys are a reliable tool for discovering natural and semisynthetic drugs. Herbs, natural, and chemical products represent 50% of the medications used internationally. The study conducted by Ali et al., [19], examined the variables influencing infertile women's use of traditional fertility treatments. According to the study, more than one-third (37%) of these women employed herbal medicines, and more than two-fifths referred to other women as private healers. Moreover, in another study by [20], infertility is not the only health issue that affects women. In this study, the median-aged women utilized herbs 55% of the time, while 17% went to a urological clinic without using herbs. The researchers discovered that religious ideals supported most conventional medical procedures [20].

Ayaz and Efe et al. [21] aimed to learn more about women's traditional fertility practices. They discovered that one-third of survey takers had tried these methods, and some of them had experienced negative effects as a result. Thus, it is important to inform infertile couples about using herbs. Every woman has to know which traditional therapies are safe and which ones are not [21]. Additionally, Udgiri et al. [22] compared the procedures used by tertiary hospitals in India's Vijayapura District in rural and urban areas. Infertility rates ranged from 7.6% in rural areas to 8.8% in urban slums. Compared to secondary infertility, primary infertility was more common. The authors discovered that most people believed that past mistakes caused infertility and used nonscientific approaches to address the issue. In this study, 177 people visited places of worship, 20 wore threads, 49 sought astrological advice, and 62 performed rituals [22].

Abolfotouh et al. [23] discovered insufficient general understanding among 59% of participants in a study conducted in Saudi Arabia. Furthermore, 76% of infertile participants demonstrated neutral conduct. There are numerous myths about the reasons for infertility. This survey discovered that 58.8% of participants blamed the problem on supernatural causes, 67.5% on black magic, 71.3% on intrauterine devices, and 42.9% on contraceptive pills. It was further discovered that in infertility situations, traditional healers were reported as the preferred choice (6.7%) [23], unlike the study by Jaradat and Zaid [8], which revealed how healers solely used (several types of) plants to cure infertility [8].

Consequently, a study surveyed 430 women; 80% were in their forties and fifties. According to the author, 25% of participants employed traditional herbal medicines to improve their fertility. Furthermore, 32% sought medical advice before beginning to use herbs. This investigation also revealed that delayed conception endangers the lives of Arab couples. Furthermore, according to the author, herbal medicines are becoming more popular in curing fertility issues [14]. In addition, Kashani et al. [24] conducted a study in Iran among infertile couples to validate the usage of herbal medicines. It was evaluated that 47.3% of female participants were well-versed in such substances. In addition, 43.4% of individuals with significant reproductive concerns were seen favorably by these standard therapies. Thirty-one percent of those who took part used herbal therapies. Only 3.2% did so after speaking with a doctor. Furthermore, 3.5% of individuals used these substances due to a lack of education [24].

## 3. Materials and Methods

A quantitative cross-sectional approach was used. The study questions were addressed using this design, establishing the correlations between the variables.

### 3.1. Ethical Approval

Approval for the study was granted by the ethical committee of the Faculty of Nursing at King Abdulaziz University in Jeddah (NREC Serial No: Ref No 2M. 72) on 18.04.2021. The permission obtained by MOH approval through Albaha Health Affairs forwarded to the selected sittings (1442-1895772/22-10-1442) on (03.06.2021g). A written informed consent form from each participant was obtained. All the participants' personal information was kept confidential and secure upon data collection.

### 3.2. Study Setting

The study was conducted in the maternity outpatient departments of the following Saudi Arabian hospitals. First was the Prince Mishari bin Sauod Hospital at Baljurashi, Albaha, outpatient gynecology department. This department has three primary clinics that maternity specialist doctors and nurses maintain. Only infertile women were chosen from all patients. The second was the infertility clinic in the outpatient department of King Fahad Hospital, Albaha. Private healers who treat infertile women based on their experience agreed to have the study conducted in their homes. Third was the gynecology outpatient department at Alaqiq General Hospital in Alaqiq, Albaha. Moreover, the fourth was the Alshefaa Medical Center, Albaha, through the gynecology outpatient department.

### 3.3. Study Period

Data were collected from 30 May 2021 until the end of January 2022.

### 3.4. Study Sampling and Sample Size

The earlier indicated strategy was used to recruit a convenience-based sample of 251 infertile women.

Table 1 shows the distributions of data collection samples according to the area collected. The infertile women involved are equal and less than 50 years old, with primary or secondary infertility related to personal or male factors. The study did not include women with medical or surgical diseases, women taking contraceptive pills, hormonal therapy, or psychotropic medications, or women with no current sexual relationship.

### 3.5. Data Collection Tool

The researcher developed a self-administered questionnaire by reviewing information about treatment practices between women and traditional healers in Albaha City. The questionnaire featured four sections to assess and evaluate these practices while collecting information about sociodemographic traits and fertility history and traditional practices used by women and their practice satisfaction.

#### 3.5.1. Part: 1. Sociodemographic Characteristics

The first section of the study obtained key demographic data from subjects who included their age, height, weight, and BMI alongside educational background, employment status, income, and residential location. The questionnaire included questions about subjects' obstetric past which encompassed marriage age and span and the counts of marriages along with pregnancies and deliveries and offspring.

#### 3.5.2. Part: 2. Fertility History

The section consisted of inquiries regarding infertility causes and whether women or men were responsible together with questions about the length of infertility exceeding 6 months. The survey investigated whether participants got gynecologist consultations along with their testing for blood tests or ultrasounds as medical approaches.

#### 3.5.3. Part: 3. Knowledge of Infertile Women About Traditional Practices

This part describes and explores traditional practices, including the following three main sections.

##### 3.5.3.1. Section I

This section concerned the participants' knowledge of traditional treatments, such as the risks and benefits of herbal treatment. This section consisted of seven items designed to determine how the participants know about traditional healers, such as through personal searches, social media, family members, or friends. This section also collected data on the practices of traditional healers and those who had advised the participants to visit a traditional healer, such as through personal search, social media, family members, or friends. Other data included the duration of infertility before a traditional healer was visited, the number of visits to traditional healers, and the treatment, such as liquid, powder, oil, ointment, abdominal massage, seeds, vaginal suppositories, or others.

##### 3.5.3.2. Section II

This section concerns the treatment prescribed to treat infertility and the duration of use. Eighteen items of traditional treatment were included as follows: powder, royal jelly, black cumin, fenugreek, myrrh, asafetida, Boswellia, green lote leaf, cumin, marjoram, olive oil, garden cress, sage, Indian costus, chamomile, pineapple, dark chocolate, and walnut. The data included duration and method of use, such as early morning on an empty stomach or at bedtime. The participants were also asked to indicate whether the prescribed treatments were mixed with other substances, including more than one treatment.

##### 3.5.3.3. Section III

This section concerns the duration of traditional treatments. This section included two points:

1. Duration of traditional treatment (month, 2 months, or three or more months);
2. Whether the infertile woman used this treatment in addition to medicine prescribed by a physician.

#### 3.5.4. Part IV: Satisfaction of Infertile Women After Using Traditional Practices

All the data collected in this part concerned traditional treatment by a traditional healer, according to the following five points:

1. Whether the woman became pregnant;
2. Duration until becoming pregnant after using a traditional treatment;
3. Side effects such as abortion, loss of weight, vaginal bleeding, irregular menstruation, or others;
4. Level of satisfaction;
5. Whether the participant planned to visit a traditional healer again.

### 3.6. Validity and Reliability of the Instrument

#### 3.6.1. Instrument Validity

In this study, the content of the questionnaire was validated using three processes:

1. Two academic experts in maternal and child nursing in the Faculty of Nursing at King Abdulaziz University;
2. Two gynecologists at Prince Mishari Hospital, Albaha;
3. An expert in statistics.

Based on the results, necessary modifications were made to the questionnaire. The experts expressed their opinions about the clarity, ease, simplicity, and comprehensiveness of the instrument's items, domains, and statements.

#### 3.6.2. Instrument Reliability

The reliability of the questionnaire was evaluated using this technique between each field and the instrument's field means. The domain “treatment with herbals” had a Cronbach's alpha of 0.702. This result was regarded as favorable, with a score of 0.813, or extremely good. Cronbach's alpha for “satisfaction level after using traditional medicine” was calculated. The overall instrument's Cronbach's alpha score was 0.74, deemed acceptable.

### 3.7. Pilot Test Instrument

Ten percent of the total study participants, 25 of whom were infertile women, from a total sample of 251 infertile women were involved in a pilot study and included in the study sample. No modification was done based on the pilot study. The participants in the pilot study were included in the main study.

### 3.8. Data Analysis

The collected data were coded, categorized based on scientific references into three categories (i.e., harmless practices, harmful practices, and practices unrelated to infertility treatment), and tabulated using the appropriate statistical significance tests to determine the relationships between the variables. All data were entered into the Statistical Software Package (SPSS) Version 25.0. The frequencies and percentages were used for the categorical variables, and a descriptive statistical analysis was performed to determine the frequency, mean, and standard deviation. A chi-squared test examined the significance between the sociodemographic data and the dependent variables (type of infertile women according to traditional practice usage). The significance was *p* ≤ 0.05. Finally, the study and control groups were compared to identify the effects of traditional practices.

## 4. Results

The results of the data analysis and the statistical interpretations of each variable based on the data collected from the questionnaire are represented in the results section. A descriptive analysis determines the sociodemographic characteristics of the study sample, and an inferential analysis was conducted to address the study questions. Simple statistics, including frequencies and percentages, were employed. The study was conducted in Albaha City on 251 infertile women diagnosed with primary or secondary infertility. The data were analyzed using SPSS Version 25.0 for data analyses. A chi-squared test was conducted to determine the relationships between the variables. A *p* value of 0.05 was considered statistically significant.

The following headings are used to present the data analysis findings.

### 4.1. Part I: The Sociodemographic Characteristics of the Study Participants

Table 2 displays the distribution of the study participants according to their sociodemographic characteristics. The study included 251 infertile women, of whom 92% were Saudi, and 8% were non-Saudi. One-half (50.6%) were aged between 30 and 39 years, and 31.9% were aged between 20 and 29 years. About half (50.6%) of the participants had bachelor's degrees, 26.3% had completed secondary school, 75.3% were housewives, 24.7% were employees, 88.4% had enough income, and 62.2% lived in urban areas.

### 4.2. Part II: The Reproductive History Data of the Study Participants

Table 3 illustrates the distribution of the study participants according to their reproductive history, 31.95% have never been pregnant, and 39.4% have no history of delivery or children.

Table 4 explains the distribution of the study participants according to their infertility history. Female factors were the reason for the delayed pregnancy (i.e., 46.6%), while (25.5%) related to both, and (27.9%) were other reasons. The longest period of late pregnancy after marriage was between one to 5 years (51.4%) and less than 1 year (29.5%). Almost all participants visited gynecologists (92.1%), while only (7.9%) had not been examined by a doctor for a delayed pregnancy.

### 4.3. Part III: Distribution of Infertile Women Who Participated in the Study According to Their Belief in Traditional Practices and Traditional Healers

**Figure 1:**
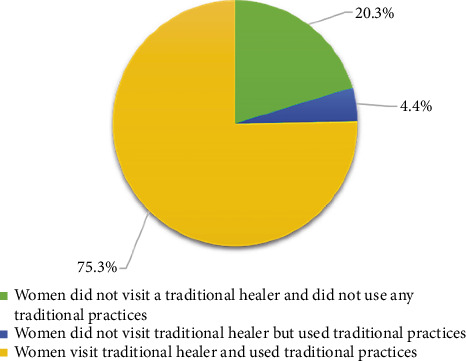
Distribution of infertile women participated in the study according to their belief in traditional practices and traditional healers (*n* = 251).

Figure 1 shows the distribution of infertile women participating in the study according to their belief in traditional practices and traditional healers: 75.3% visited traditional healers and used traditional practices, and 20.3% did not visit traditional healers and did not use any traditional practices. Only 4.4% did not visit traditional healers but used traditional practices.

### 4.4. Part IV: Knowledge and Practices of Infertile Women About Traditional Healers and Traditional Practices

Table 5 describes the women's knowledge of traditional healers and practices; 51.0% reported that it is useful, while 45.8% had no prior knowledge, and only 3.2% answered that it is harmful. A total of 39.0% of infertile women knew about traditional healers through their friends, while 22.7% knew about them through social media. Regarding infertile women who visited the level of education of the traditional healers, 76.2% were traditional healers based on experience, and 9. % were doctors.

Table 6 shows the distributions of women according to the time of the first visit of a traditional healer and quantity of visits: 50.3% reported that the period after which the traditional healer was visited since the beginning of the marriage was from one to 5 years, followed by from 6 months to 1 year (34.9%); 47.6% had visited a traditional healer three or more times, 30.2% had visited a traditional healer only once, and 22.2% had visited a traditional healer twice.

Table 7 proves the traditional treatment prescribed by the traditional healer to treat infertile women. Herbs and abdominal massages were the most given treatment (63.5%). Treatments with drinks and oils were around 20.1% each; vaginal suppositories were 4.2%. Vegetable grains and seeds were 6.8%, and ointments were only 1.0%.

Table 8 presents the study participants' practices. One month was chosen by 47.5% of participants as the length of time for traditional treatment use, followed by 2 months by 20.0% and 3 months or more by 32.5%. Furthermore, just 28% of participants reported that they combined medical medicine with traditional medicine.

### 4.5. Part V: Distribution of Study Participants According to Benefits, Side Effects, and Level of Satisfaction After Employing of Traditional Practices

Table 9 explains the study participants' distribution according to benefits and side effects after using traditional practices. More than one-third (39.5%) became pregnant after using traditional therapy, while more than half (60.5%) reported no pregnancy accrued after using traditional treatment; 29.0% became pregnant in less than 6 months after using traditional methods; and 11.0% became pregnant between 6 months and 1 year after using traditional methods. Moreover, 65.5% had no side effects after using traditional therapy. Furthermore, 12.5% lost weight, 8% recorded irregular menstruation, and 3.0% had adoration. In addition, 45.5% planned to visit a traditional healer again, while 54.5% had no plan to revisit a traditional healer.

**Figure 2:**
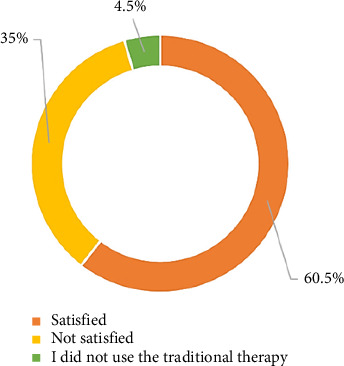
Distribution of study participants according to the level of satisfaction after using of traditional practices (*n* = 200).

Figure 2 illustrates the distribution of the study participants according to their level of satisfaction after using traditional practices. More than half (60.5%) of the participants were satisfied with their experiences, while more than one-third (35%) were unsatisfied.

### 4.6. Part VI: Distribution of Traditional Treatment According to Usage Details and Its Effect: Harmful, Harmless or Have No Relationship With Infertility

Table 10 shows the distribution of traditional treatments according to usage and their effects on infertility. Participants implemented various herbs and massage practices to determine the effectiveness of the treatment or side effects. Herbal treatment showed both results, i.e., beneficial for some participants as they believed, and some participants had side effects after employing herbal practices. Based on the current study result, the researcher found that royal jelly, black cumin, fenugreek, myrrh, marjoram, garden cress, and walnut are highly safe herbs, while green lote leaves and Boswellia have to be excluded from infertility treatment for not being safe evidence by their statistical significance with the side effects (*p* < 0.05).

### 4.7. Part VIII: The Relationship Between Herb Consumption and the Occurrence of Pregnancy After Using Traditional Therapy

Table 11 presents the relationship between herb consumption and pregnancy after using traditional therapy, with no statistical significance with all used herbs and occurrence of pregnancy after using traditional therapy (*p* > 0.05).

### 4.8. Part IX: The Relationship Between Herb Consumption and Side Effects

As indicated in Table 12, there are relationships between herb consumption and side effects. The results showed that two herbs (green lote leaves and Boswellia) had statistically significant side effects (*p* < 0.05). The percentages of herbs that showed a negative side effect ranged between 8.5% and 13.0%. The percentages of negative side effects of unused herbs ranged between 39.0% and 34.5%. The remaining herbs did not cause side effects.

### 4.9. Part X: The Relationship Between Abdominal Massage as an Infertility Treatment Method Used by Study Participants and Pregnancy and Side Effects

Table 13 shows the conformity of the relationships between abdominal massage as an infertility treatment method, pregnancy, and side effects. The results showed that 12% of the women who used abdominal massage had side effects. The results showed that abdominal massage did not have a statistical significance with pregnancy occurrence after traditional therapy (*p* > 0.05).

## 5. Discussion

This research analyzed the native practices which infertile women in Albaha City Saudi Arabia apply to cure infertility. Data generated from this research study match existing literature to create a whole picture about traditional treatment methods for infertility.

Experts have identified infertility as a major health problem which occurs in similar proportions among men and women throughout the world. The number of infertile couples together with their corresponding traditional treatment methods differ extensively between different population areas. Traditional medicine serves as the primary approach for treating infertility among Saudi citizens who continue showing interest in its results. Pregnant women frequently took herbs according to Zaitoun et al. [25] although there was insufficient evidence about their safety. Traditional therapies proved ineffective for conception according to 60.5% of participants, but 39.5% succeeded in getting pregnant. The results demonstrate that traditional treatments generated successful pregnancies within 6 months or 1 year after the start of therapy in 29.0% and 11.0% of subjects accordingly [25].

The present research examines *Apis mellifera* (royal jelly) usage as an everyday herbal remedy for infertility treatment. Most research participants consumed royal jelly on an empty stomach either in the morning or before going to bed since they thought this practice would boost their fertility capabilities. The research results match those presented by Chalapathy et al. [26] about royal jelly's capability to rectify hormonal irregularities and enhance fertility. The research by Chalapathy et al. [26] demonstrated royal jelly functions as a successful ingredient supporting infertility treatment. Research indicates that royal jelly does not relate to pregnancy outcomes although it remains popular among users, thus requiring additional studies to prove its effectiveness in treating infertility.

The participants in this study engaged in black cumin (Nigella sativa) usage which confirmed previous findings documented by Djerrou et al. [27] who recognized black cumin as an infertile treatment herb. Participants who used black cumin products experienced no meaningful adverse effects during the study, and most participants reported higher chances of getting pregnant. Results indicate that black cumin poses no safety risks for participants according to the data assessment like the findings of Djerrou et al. [27]. Royal jelly received the same treatment as black cumin regarding pregnancy outcomes because no statistical relationship emerged between use and results although many people believe in its effectiveness, but more clinical research is needed.

Participants regularly used Trigonella Foenumgraecum (fenugreek) to achieve their pregnancy goals. Study participants thought fenugreek could boost conception opportunities, but this research showed no meaningful connection between fenugreek use and pregnancy results. Research by El-Hak and Elrayess [28] indicated that fenugreek provides reproductive health benefits, but their study along with others showed that clinical evidence supports the effectiveness of fenugreek for infertility treatment remains limited according to this study's findings. Commiphora Molmol Engler (Myrrh) proved to be a widely used remedy for infertility treatment according to the results of this study. The research revealed no meaningful link between pregnant outcomes and the use of myrrh by women. Research by Wal et al. [29] listed myrrh among herbs used for reproductive health although their study did not identify enough evidence on its ability to treat infertility.

The current research established marjoram (Origanum majorana) herb as one of its primary findings. Marjoram received widespread acceptance from the research participants as an effective herb for treating infertility, while they considered it a safe natural medicine. The same effect has been reported by El-Wakf et al. [30] during their research on marjoram oil treatment of infertility in animals. Research findings presented in this study indicate the potential value of marjoram as an infertility treatment addition because it offers a safe option.

Participants from the study used Lepidium sativum (garden cress) for infertility cases as reported by Prajapati and Dave [31]. The study results show that marjoram does not work as an infertility treatment despite widespread application for this purpose. Research shows garden cress seeds are present in Indian traditional healing systems according to Prajapati and Dave [31], but specific evidence about their effectiveness for infertility is still inconclusive based on this study results.

A large portion of 24% women from this study relied on walnut (Juglans regia) to enhance their fertility according to their beliefs. The researchers at Özkan et al. [28] discovered that walnut consumption creates favorable effects on ovulation and fertility. This study confirms that walnut serves as one of the widely used traditional fertility remedies, yet additional research must demonstrate its particular function in fertility treatment.

The research study concluded that Boswellia Sacra Flueck (Boswellia) as well as Zizyphus Spina-Christi (green lote leaves) were ineffective herbal treatments which may present risks to fertility health. Research by Abdelhalim and Saleem [13] and Zaatout [14] found negative aspects regarding these herbs since Boswellia affected menstrual periods yet failed to boost reproductive health. The research demonstrated that green lotus leaves have a strong statistical link to negative side effects indicating potential damage to patients receiving infertility treatment.

Participating women used abdominal massage extensively as an accepted traditional treatment for infertility. The study results showed no statistical connection between pregnancy results from using abdominal massage as a treatment. Research findings like these parallel previous studies which warn about risks from abdominal massage including genital organ dislocation together with bleeding and miscarriage mainly when people unknowingly use it during pregnancy [11]. Additional research should investigate the security level and effectiveness of abdominal massage applications for infertility treatment.

### 5.1. Limitations

Several restrictions can be identified in this research project. The outcomes from this study became less transferable because researchers used only 251 participants as their sample. An enlargement of sample size together with various demographic representation in the population will yield more comprehensive findings about infertility treatments. The study analyzed infertile women exclusively; thus, it limited the comprehension of gender-specific infertility practices. There were fewer than half of the participants who maintained their treatment at gynecology clinics because such extended follow-ups were excluded from the research. The process of ethical approval together with the refusal of multiple traditional healers to participate resulted in reduced information about traditional practices reached by the researchers.

### 5.2. Strengths

One key strength of this study is its focus on traditional practices for treating female infertility in Saudi Arabia, an area that has been under-researched. This study provides valuable insights into the benefits and risks of traditional treatments, offering a unique perspective on the intersection of traditional and modern medical practices. It is the first study to comprehensively explore traditional infertility practices in this region, contributing important knowledge to the field.

### 5.3. Recommendations

Healthcare providers should offer emotional support to infertile couples, helping them maintain a positive outlook. Access to advanced infertility treatments should be improved to ensure better outcomes. A larger scale study with a more diverse sample is needed to enhance the generalizability of the results. Healthcare professionals should be educated about traditional practices and their potential risks. Future research should focus on the safety and efficacy of commonly used herbal remedies. An educational campaign to raise awareness about unsafe practices, especially during pregnancy, is crucial. Finally, integrating evidence-based traditional treatments into formal healthcare systems could offer safer, more affordable options for those seeking traditional therapies.

## 6. Conclusion

The research presents important information about the customary infertility treatments which citizens of Albaha City Saudi Arabia have adopted. The investigation shows that traditional remedies remain commonly used for infertility treatment through herbs and stomach massages, thereby demanding more scientific research about their capabilities. Healthcare providers should maintain cultural sensitivity during their work to support evidence-based approaches for infertility care. This research advances our knowledge about traditional approaches to infertility treatment, thus indicating that upcoming clinical investigations will shape well-researched and dependable professional practices.

## Figures and Tables

**Table 1:** Distribution of data collection sample according to study setting (n = 251).

Total number of hospitals/regions	Population	Study sample
Prince Mishari Hospital	404	83
King Fahad Hospital	585	62
Traditional healers	No statistics available	88
Alaqiq General Hospital	No statistics available	6
Alshefaa Medical Center	Around 30 per month	12

**Table 2:** Distribution of the study participants according to their sociodemographic characteristics (*n* = 251).

Sociodemographic characteristics	*N*	%
Age		
Less than 20 years	6	2.4
20–29 years	80	31.9
30–39 years	127	50.6
40 years and more	38	15.1
Nationality		
Saudi	231	92.0
Non-Saudi	20	8.0
Education level		
Reads and writes	11	4.4
Primary school	14	5.6
Middle school	24	9.6
Secondary school	66	26.3
University level	127	50.6
Postgraduate	9	3.6
Occupation		
House wife	189	75.3
Employee	64	24.7
Monthly income		
Enough	222	88.4
Not enough	29	11.6
Region		
Urban area	156	62.2
Rural area	95	37.8

**Table 3:** Distribution of the study participants according to their reproductive history (*n* = 251).

Reproductive history	*N*	%
Number of pregnancies		
None	80	31.9
1	56	22.3
2	48	19.1
3 and more	67	26.7
Number of deliveries		
None	99	39.4
1	51	20.3
2	45	17.9
3 and more	56	22.3
Number of abortions		
None	175	69.7
1	38	15.1
2	24	9.6
3 and more	14	5.6
Number of kids		
None	99	39.4
1	50	19.9
2	47	18.7
3 and more	55	21.9

**Table 4:** Distribution of the study participants according to their infertility history (*n* = 251).

Infertility history	*N*	%
Reasons for delayed pregnancies		
Female factors	117	46.6
Both	64	25.5
Unknown causes	70	27.9
Time of the first pregnancy after marriage		
From 6 months to ≤ 1 year	74	29.5
> One year to ≤ 5 years	129	51.4
> 5 years to ≤ 10 years	40	15.9
> 10 years to ≤ 15 years	5	2.0
More than 15 years	3	1.2
Do you visit the gynecologists		
Yes	231	92.1
No	20	7.9
Have you done medical examinations regarding the delayed pregnancy?^∗^		
Blood investigations	186	74.1
Vaginal or abdominal ultrasound	176	70.1
Semen test	19	7.6
Other	17	6.8

^∗^More than one answer.

**Table 5:** Knowledge of women about traditional healers and traditional practices.

Knowledge about traditional healers and traditional practices	*N*	%
Traditional therapy is used for infertility (*n* = 251)		
Useful	128	51
Harmful	8	3.2
I have no prior knowledge	115	45.8
How did you know about the traditional healer? ^∗^ (*n* = 189)		
Personal search	50	26.4
Social media	43	22.7
Via friend	74	39.0
Forced by a relative	4	2.1
Through husband's family	15	8.0
Wife's family	11	5.8
Others	10	5.2
Education level of a traditional healer (*n* = 189)		
Doctor	17	9.0
Nurse	2	1.0
Traditional healers	144	76.2
I do not know	25	13.3
Others	1	0.5

^∗^More than one Answer.

**Table 6:** Distributions of women according to the time of first visit of traditional healer and the number of visits (*n* = 189).

Time of first visit of a traditional healer and number of visits	*N*	%
The period from marriage and first visit of a traditional healer		
From 6 months to 1 year	66	34.9
From 1 to 5 years	95	50.3
From 5 to 10 years	18	9.5
From 10 to 15 years	9	4.8
From 15 to 20 years	1	0.5
Number of times a traditional healer was visited		
Once	57	30.2
Twice	42	22.2
Three and more	90	47.6

**Table 7:** Traditional treatment prescribed by the traditional healer to treat infertility (*n* = 189).

Traditional treatment prescribed by a traditional healer	*N*	%
Treatment^∗^		
Drinks	38	20.1
Herbs	120	63.5
Oils	38	20.1
Ointments	2	1.0
Abdominal massage	120	63.5
Vegetable grains and seeds	13	6.8
Vaginal suppositories	8	4.2
Others	2	1.0

^∗^More than one answer.

**Table 8:** Practices of study participants for the traditional treatment, duration, and mode of use (*n* = 200).

Practices of traditional treatment, duration, and mode of use	*N*	%
Duration of traditional treatment use		
One month	95	47.5
Two months	40	20.0
Three months and above	65	32.5
Was traditional medicine used with medical medicine?		
Yes	56	28.0
No	144	72.0

**Table 9:** Distribution of traditional treatment according to usage details and its effect: harmful, harmless, or have no relationship with infertility (*n* = 200).

Benefits and side effects after traditional practices' implementation	*N*	%
Did you get pregnant after using traditional therapy?		
Yes	79	39.5
No	121	60.5
The duration during which pregnancy accrued		
6 months and less	58	29.0
> 6 months–≤ one year	22	11.0
> 1 year–2 years	8	4.0
More than 2 years	5	2.5
No pregnancy accrued until now	107	53.5
Side effects after using traditional therapy ^∗^		
Abortion	6	3.0
Loss of weight	25	12.5
Vaginal bleeding	3	1.5
Irregular menstruation	16	8.0
Others	24	12.0
No side effects	131	65.5
Do you have the plan to visit the traditional healer again		
Yes	91	45.5
No	109	54.5

^∗^More than one answer.

**Table 10:** Distribution of traditional treatment according to usage details its effect: harmful, harmless or have no relationship with infertility (*n* = 200).

S. No.	Herbs	Administration and dosage	*N*	%	Category
1	Scientific name: unknown English name: powder Local name: مسحوق من الأعشاب	Mix it with water and drink	76	38.0	Harmful Its unknown content, prescribed by a traditional healer, having low evidence and is not safe, especially during pregnancy
		Mix it with honey and drink	62	31.0	
		Certain amounts are to be taken directly	4	2.0	
		Mix with other components (marjoram, anise, sage, and milk)	3	1.5	
	Time of use	Morning drink on an empty stomach	126	63.0	
		Drink in the evening before bed	19	9.5	
		Apply on the evening before bed	0	0.0	
		Others	0	0.0	

2	Scientific name: *Apis mellifera* L English name: Royal Jelly Local name:غذاء ملكات النحل	Mix it with water and drink	81	40.5	Harmless treat infertility related to imbalanced hormones can increase the parameters of the genital in certain sperm amount and motility
		Mix it with honey and drink	20	10.0	
		Certain amounts are to be taken directly	6	3.0	
	Time of use	Morning drink on an empty stomach	101	50.5	
		Drink in the evening before bed	4	2.0	
		Apply on the evening before bed	1	0.5	
		Others	1	0.5	

3	Scientific name: *Nigella sativa* L English name: black cumin Local name: الحبة السوداء	To be mixed with other substances (honey, olive, water, date, royal jelly, fenugreek, green lote leaves, and cinnamon)	65	32.5	Harmless manage the disorders of infertility in both males and females used actively to seek treatment and polycystic ovarian syndrome therapy in women to control the cholesterol level and HDL levels that are unregulated and related to PCOS
		Soak and drink the soaked	16	8.0	
		Crush and then certain amount has to be taken	24	12.0	
		Mix with oil to be applied on the body	10	5.0	
	Time of use	Morning drink on an empty stomach	70	35.0	
		Drink in the evening before bed	19	9.5	
		Apply on the evening before bed	9	4.5	
		Others	17	32.5	

4	Scientific name: *Trigonella foenum raecum* L English name: fenugreek Local name: الحلبة	To be mixed with another substance (date, honey, and milk)	49	24.5	Harmless enlarged the physiological side of libido in healthy adult men. Enlarged total levels of testosterone, sexual purpose No studies ensure that it considers anti-fertility among women
		Soak and drink the soaked	35	17.5	
		Crush and then certain amount has to be taken	12	6.0	
		Mix with oil to be applied on the body	4	2.0	
	Time	Morning drink on an empty stomach	61	30.5	
		Drink in the evening before bed	20	10.0	
		Apply on the evening before bed	8	4.0	
		Others	11	5.5	

5	Scientific name: *Commiphora molmol* L English name: Myrrh Local name: المرة	To be mixed with another substance (honey, lump sugar, and herbs)	40	20.0	Harmless It was used to treat Menorrhagia as one of the symptoms related to PCOS. used to stimulate the menstrual gush, uterus disinfectant after giving birth and steamy cleansing for vaginal virus
		Soak and drink the soaked	22	11.0	
		Crush and then certain amount has to be taken	9	4.5	
		Mix with oil for the body	5	2.5	
	Time of use	Morning drink on an empty stomach	48	24.0	
		Drink in the evening before bed	14	7.0	
		Apply on the evening before bed	7	3.5	
		Others	7	3.5	

6	Scientific name: *Boswellia Sacra* L English name: Boswellia Local name: لبان الذكر	To be mixed with another substance	16	8.0	Harmful The current study shows statistical significant with side effects recorded by participants after using the traditional treatment
		Soak and drink the soaked	27	13.5	
		Crush then certain amount to be taken	6	3.0	
		Mix with oil to be applied on the body	5	2.5	
	Time of use	Morning drink on an empty stomach	28	14.0	
		Drink in the evening before bed	8	4.0	
		Apply on the evening before bed	12	6.0	
		Others	6	3.0	

7	Scientific name: *Zizyphus Spina-Christi* English name: Green lote leaves Local name: ورق السدر	To be mixed with another substance (honey, olive, and cinnamon)	22	11.0	Harmful The current study shows statistical significance with side effects recorded by participants after using the traditional treatment
		Soak and drink the soaked	12	6.0	
		Crush then certain amount to be taken	15	7.5	
		Mix with oil to be applied on the body	23	11.5	
	Time of use	Morning drink on an empty stomach	23	11.5	
		Drink in the evening before bed	11	5.5	
		Apply on the evening before bed	30	15.0	
		Others	8	4.0	

8	Scientific name: *Origanum majorana* English name: Marjoram Local name: البردقوش	To be mixed with another substance (sage)	21	10.5	Harmless Used for reduction of testicular lipid accumulation, elevate the sperm amount
		Soak and drink the soaked	67	33.5	
		Crush then certain amount to be taken	9	4.5	
		Mix with oil to be applied on the body	3	1.5	
	Time of use	Morning drink on an empty stomach	39	19.5	
		Drink in the evening before bed	19	9.5	
		Apply on the evening before bed	7	3.5	
		Others	35	17.5	

9	Scientific name: *Lepidium saliva* L English name: Garden Cress Local name: حب الرشاد	With date	61	30.5	Harmless It could improve the histoarchitecture of rabbit testis and may be used to recover the fertility in rabbits. It is improving semen excellence and safe the sperm of men
		To be mixed with another substance and eaten (lignum seeds, fenugreek, cumin, honey, black cumin, and marjoram)	15	7.5	
		To be mixed with another substance and drink (milk)	4	2.0	
	Time of use	Morning drink on an empty stomach	63	31.5	
		Drink in the evening before bed	15	7.5	
		Apply on the evening before bed	0	0.0	
		Others	2	1.0	

10	Scientific name: *Juglans regia* L English name: walnut local Name: الجوز/عين الجمل	To be mixed with another substance (nuts, dates, and honey)	16	8.0	Harmless Evidence has been approved to enhance the chances of pregnancy when infertility is related to male factors
		Soak and drink the soaked	8	4.0	
		Crush and then certain amount has to be taken	14	7.0	
		Mix with oil for the body	1	0.5	
	Time of use	Morning drink on an empty stomach	12	6.0	
		Drink in the evening before bed	14	7.0	
		Apply on the evening before bed	2	1.0	
		Others	11	5.5	

**Table 11:** The relationship between herb consumption and occurrence of pregnancy after using traditional therapy (n = 200).

Herbs	Did you get pregnant after using traditional therapy?						*X* ^2^	*p* value
	Yes		No		Total			
	*N*	%	*N*	%	*N*	%		
Powder								
Used	58	29.0	87	43.5	145	72.5	0.055 ns	0.814
Not used	21	10.5	34	17.0	55	27.5		
Royal jelly								
Used	43	21.5	64	32.0	107	53.5	0.045 ns	0.831
Not used	36	18.0	57	28.5	93	46.5		
Black cumin								
Used	49	24.5	66	33.0	115	57.5	1.094 ns	0.296
Not used	30	15.0	55	27.5	85	42.5		
Fenugreek								
Used	42	21.0	58	29.0	100	50.0	0.523 ns	0.470
Not used	37	18.5	63	31.5	100	50.0		
Myrrh								
Used	27	13.5	49	24.5	76	38.0	0.810 ns	0.368
Not used	52	26.0	72	36.0	124	62.0		
Not used	61	30.5	82	41.0	143	71.5		
Boswellia								
Used	19	9.5	35	17.5	54	27.0	0.576 ns	0.448
Not used	60	30.0	86	43.0	146	73.0		
Green lote leaves								
Used	26	13.0	46	23.0	72	36.0	0.541 ns	0.462
Not used	53	26.5	75	37.5	128	64.0		
Not used	51	25.5	73	36.5	124	62.0		
Marjoram								
Used	39	19.5	61	30.5	100	50.0	0.021 ns	0.885
Not used	40	20.0	60	30.0	100	50.0		
Not used	46	23.0	60	30.0	106	53.0		
Garden cress								
Used	31	15.5	49	24.5	80	40.0	0.031 ns	0.859
Not used	48	24.0	72	36.0	120	60.0		
Not used	71	35.5	110	55.0	181	90.5		
Walnut								
Used	14	7.0	25	12.5	39	19.5	0.263 ns	0.608
Not used	65	32.5	96	48.0	161	80.5		

*Note:X* ^2^ = chi-squared.

Abbreviations: ns = not significant, Na = not available.

^∗^≤ 0.05.

^∗∗^≤ 0.01.

^∗∗∗^≤ 0.001.

**Table 12:** The relationship between herb consumption and side effects (n = 200).

Herbs	Side effects						*X* ^2^	*p* value
	No		Yes		Total			
	*N*	%	*N*	%	*N*	%		
Powder								
Used	63	31.5	45	22.5	108	72.5	1.313 ns	0.252
Not used	32	16.0	15	7.5	47	27.5		
Royal jelly								
Used	47	23.5	32	16.0	79	53.5	0.219 ns	0.640
Not used	48	24.0	28	14.0	76	46.5		
Black cumin								
Used	53	26.5	36	18.0	89	57.5	0.267 ns	0.606
Not used	42	21.0	24	12.0	66	42.5		
Fenugreek								
Used	46	23.0	29	14.5	75	50.0	0.000 ns	0.992
Not used	49	24.5	31	15.5	80	50.0		
Myrrh								
Used	35	17.5	20	10.0	55	38.0	0.198 ns	0.657
Not used	60	30.0	40	20.0	100	62.0		
Not used	77	38.5	41	20.5	118	71.5		
Boswellia								
Used	17	8.5	20	10.0	37	27.0	4.823	0.028^∗^
Not used	78	39.0	40	20.0	118	73.0		
Green lote leaves								
Used	26	13.0	26	13.0	52	36.0	4.204	0.040^∗^
Not used	69	34.5	34	17.0	103	64.0		
Not used	61	30.5	39	19.0	100	62.0		
Marjoram								
Used	47	23.5	30	15.0	77	50.0	0.004 ns	0.949
Not used	48	24.0	30	15.0	78	50.0		
Not used	57	28.5	31	15.5	88	53.0		
Garden cress								
Used	35	17.5	26	13.0	61	40.0	0.649 ns	0.420
Not used	60	30.0	34	17.0	94	60.0		
Not used	85	42.5	56	28.0	141	90.5		
Walnut								
Used	16	8.0	9	4.5	25	19.5	0.092	0.761
Not used	79	39.5	51	25.5	130	80.5		

*Note:X* ^2^ = chi-squared.

Abbreviations: ns = not significant, Na = not available.

^∗^≤ 0.05.

^∗∗^≤ 0.01.

^∗∗∗^≤ 0.001.

**Table 13:** The relationship between abdominal massage as an infertility treatment method used by study participants, pregnancy and side effects (n = 146).

	Abdominal massage						*X* ^2^	*p* value
	Yes		No		Total			
	Count	%	Count	%	Count	%		
Did you get pregnant after using traditional therapy?								
Yes	34	17.0	26	13.0	60	39.5	0.211	0.646
No	52	26.0	34	17.0	86	60.5		
Side effects								
No	62	31.0	28	14.0	90	47.5	9.664	0.002^∗^
Yes	24	12.0	32	16.0	56	30.0		

^∗^ *p* value > 0.05.

## Data Availability

The data supporting the findings of this study are available from the corresponding author upon reasonable request.
